# Public Perceptions of Climate Change and Its Health Impacts: Taking Account of People’s Exposure to Floods and Air Pollution

**DOI:** 10.3390/ijerph19042246

**Published:** 2022-02-16

**Authors:** Hilary Graham, Alexander Harrison, Pete Lampard

**Affiliations:** Department of Health Sciences, University of York, York YO10 5DD, UK; alexander.harrison@york.ac.uk (A.H.); pete.lampard@york.ac.uk (P.L.)

**Keywords:** climate change, public perceptions, climate-related exposures, flood, air pollution

## Abstract

Climate change-related exposures such as flooding and ambient air pollution place people’s health at risk. A representative UK survey of adults investigated associations between reported flooding and air pollution (in the participants’ local area, by the participant personally, and/or by family and close friends) and climate change concerns (CCC) and perceptions of its health impacts (PIH). In regression analyses controlling for socio-demographic factors and health status, exposure was associated with greater CCC and more negative PIH. Compared to those with low CCC, participants who reported local-area exposure were significantly more likely to be fairly (OR 2.07, 95%CI 1.26, 3.40) or very concerned (OR 3.40, 95%CI 2.02, 5.71). Odds of greater CCC were higher for those reporting personal and/or family exposure (‘fairly concerned’: OR 2.83, 95%CI 1.20, 6.66; ‘very concerned’: OR 4.11, 95%CI 1.69, 10.05) and for those reporting both local and personal/family exposure (‘fairly concerned’: OR 3.35, 95%CI 1.99, 5.63; ‘very concerned’: OR 6.17, 95%CI 3.61, 10.55). For PIH, local exposure significantly increased the odds of perceiving impacts as ‘more bad than good’ (1.86, 95%CI 1.22, 2.82) or ‘entirely bad’ (OR 1.88; 95%CI 1.13, 3.13). Our study suggests that public awareness of climate-related exposures in their local area, together with personal exposures and those of significant others, are associated with heightened concern about climate change and its health impacts.

## 1. Introduction

The world is warming very quickly, driven by the upward trend in greenhouse gas (GHG) emissions [1,2,3]. Rising global temperatures are increasing the incidence and severity of extreme weather events, with greater population exposure to flooding across western and central Europe [4,5]. GHG emissions are also the major source of poor air quality, both with respect to fine particulate matter (PM_2.5_) and other air pollutants released during the combustion of fossil fuels for power generation, residential and commercial energy use, and transport. Flooding [6,7] and air pollution [8,9,10,11] are placing human health at risk. In the UK, the 12 months up to July 2021 included storms and resultant severe flooding in August, November, and December 2020 as well as flash floods in January, June, and July 2021 [12,13]. UK annual monitoring data [14] indicate that 28% of local areas fail to meet the 2005 WHO guidelines on air quality [9]; the more stringent thresholds in the WHO’s updated guidelines are likely to increase this proportion [15]. 

Studies of populations exposed to flooding and air pollution have reported participants’ concerns about climate change and its adverse impacts [16,17,18]. However, little is known about whether exposure is a predictor of climate change concern (CCC) and perceptions of its health impacts (PIH) in the general population. A 2018 review of studies of public perceptions of the health impacts of climate change did not report on the studies’ inclusion of measures of exposure [19]. A more recent review [20] found two studies that included UK populations [21,22], neither of which investigated exposures as potential factors predicting climate change concern and perceptions of health impacts.

To address this gap, we investigate whether reported exposure to floods and air pollution is associated with greater concern about climate change and more negative perceptions of its health impacts. We follow Strobe guidelines for reporting observational studies [23].

## 2. Materials and Methods

Data and sample profile: The study is based on an online cross-sectional survey of 1024 adults aged ≥ 18 conducted via the Qualtrics survey platform [24] in July 2021. It was approved by the Health Sciences Research Governance Committee, University of York (ref: HSRGC/2020/409/C), and informed consent was secured from study participants. The survey used quota-controlled recruitment to match the national UK population for gender, age group, ethnic group [25], educational attainment (International Standard Classification of Education—ISCED [26] and location (UK country/England region [27]). Where numbers were small, response categories were combined (age group, ethnic group, country/region). To avoid potential priming effects that participation in previous climate change research may have had on responses, participants were excluded if they had taken part in a climate-change related survey in the previous year. 

Outcome measures: Climate change concern (CCC) was measured by the question ‘How concerned, if at all, are you about climate change?’ [28], with a 4-option response (not at all concerned, not very concerned, fairly concerned, very concerned). All participants were asked this question (*n* = 1024). Perceptions of health impacts (PIH) were measured by the question ‘Overall, do you think climate change will be good or bad for the health of people in the UK?’ with a 5-option response (entirely good, more good than bad, equally good and bad, more bad than good, and entirely bad). An earlier question asked, ‘Thinking about people’s health, which of these statements best describes your views about the impacts of climate change on people’s health in the UK?’. Participants who answered ‘Climate change will never have an impact on people’s health in the UK’ were not asked the PIH question (*n* = 60; 5.9%). 

Exposure measure: At the end of the survey, participants were asked about exposure to ‘flooding’ and ‘air pollution (poor air quality)’ in the past 12 months. The question avoided terms that may elicit a strong association with climate change, for example ‘extreme weather events’ [29]. Participants were asked separately if they were aware of these exposures ‘in your local area’, if they personally experienced them, and if a family member or a close friend had experienced them. The latter two responses (personal experience and family/friend experience) were combined into a single ‘personal exposure’ category because of small numbers. 

Responses for flooding and air pollution were combined due to some correlation of exposures and small numbers in the separate levels of exposure (local/personal). This produced four categories: 1—Not exposed to either flooding or air pollution at local or personal level; 2—Local exposure to one/both of flooding and air pollution; 3—Personal exposure to one/both; 4—Local and personal exposure to one/both. 

*Analysis:* Bivariate analyses investigated associations between reported exposure and CCC and between reported exposure and PIH. Bivariate associations between exposure and socio-demographic factors (gender, age group, ethnic group, education, housing tenure, UK country/English region) and health status were also examined (response categories for these factors are summarised in Table 1). 

In two regression models, multinomial regression was used to assess associations between exposures and CCC and PIH, using SPSS version 26 [30]. The reference groups were, respectively, not being concerned about climate change (combining those not at all and not very concerned) and not perceiving the health impacts of climate change as bad (combining those perceiving the impacts as entirely good, more good than bad, and equally good and bad). 

The models were built hierarchically with socio-demographic factors and health status added before the exposure measures. The backwards stepwise approach was used; the threshold for retention was a cut-off of *p* < 0.1 for inclusion in the final model. Model testing was performed with goodness of fit, r^2^ estimates and log likelihood presented for each model along with the percentage correctly predicted.

## 3. Results 

### 3.1. Sample Profile 

The majority of participants were concerned about climate change (fairly: 47%; very: 36%) and perceived its impacts on health in the UK to be negative (more bad than good: 46%; entirely bad: 25%). With respect to exposures, flooding in their local area and/or personally (by the individual, family, close friend) was reported by 36% of participants. For air pollution, the proportion was 43%.

### 3.2. Bivariate Associations between Reported Exposure (Floods and Air Pollution) and Climate Change Concern and Perceived Health Impacts of Climate Change

As Table 2 indicates, reported exposure to flooding and air pollution was associated with both CCC and PIH (*p* < 0.001). Among those not concerned about climate change, 66% reported no exposure to air pollution and/or flooding in their local area, personally or among family and friends. Among those fairly and very concerned about climate change, the proportions were 43% and 31%, respectively. A similar association is evident with respect to PIH (*p* < 0.001). Nearly half (49%) of those who did not perceive the health impacts as bad (as entirely good, more good than bad, equally good and bad) reported no exposure to flooding or air pollution compared with 41% and 29% in the ‘more bad than good’ and ‘entirely bad’ groups. 

In the bivariate analyses (supplementary Table S1), education was significantly associated with CCC (*p* < 0.001) and PIH (*p* < 0.001), with a higher proportion of those in the highest educational group (Level 3) being very concerned about climate change and perceiving its health effects to be entirely bad. Other socio-demographic factors were significantly associated with CCC: being female (*p* < 0.01), being older (*p* < 0.001), and living in London/southern England Region (*p* < 0.001) and with PIH (housing tenure, *p* < 0.01). Health status was not significantly associated with either outcome (>0.05). 

### 3.3. Regression Analysis

*Climate change concern.* The regression model assessed the strength of association between reported exposure and CCC. It estimated the odds of being fairly concerned or very concerned about climate change compared to not being concerned (not at all concerned/not very concerned), taking account of socio-demographic factors, health status and exposure. The effects of exposure are presented in Table 3 (in full in Supplementary Table S2).

As Table 3 indicates, exposure was significantly associated with greater CCC, with exposure at local, personal, or both levels always having a positive association with concern. Compared to not being concerned about climate change, reported exposures in the local area or at a personal level (personally/among family and friends) doubled the odds of being fairly concerned (local: 2.07; 95%CI 1.26, 3.40; personal: 2.83; 95%CI 1.20, 6.66). Reporting both local and personal exposure increased the odds to 3.35 (95%CI 1.99, 5.63). Being female and younger were also associated with a greater likelihood of being fairly concerned; living outside London/Southern England reduced the odds (Table S2). 

With respect to being very concerned, personal exposure was associated with higher odds (OR 4.11; 95%CI 1.69, 10.05) than local exposure (OR 3.40; 95%CI 2.02, 5.71). The largest odds were associated with reporting both local and personal exposure (OR 6.17; 95%CI 3.61, 10.55). Being female, younger, and achieving the highest level of education (Level 3) increased the odds of being very concerned; living outside London/Southern England reduced the odds (Table S2). 

The model was a good fit with 53% of all cases correctly predicted, significant (>0.05) for the goodness of fit test, and r^2^ indicating that 18% of all variance in the level of climate concern was accounted for in this model.

#### Perceived Impact on Health

The regression model estimated the contribution of exposure to the odds of perceiving the health impacts of climate change to be negative (more bad than good or entirely bad). The reference category included all other responses (entirely good, more good than bad, equally good and bad). As above, the analysis took account of socio-demographic factors, health status, and exposure. The effects of exposure are presented in Table 4 (in full in Supplementary Table S3). 

As Table 4 indicates, local exposure is a significant predictor of perceiving the health impacts of climate change as being more bad than good (OR 1.86, 95%CI 1.22, 2.82, *p* < 0.01). Being female and having a higher level of educational attainment were also associated with a greater likelihood of perceiving the health impacts of climate change as being more bad than good (Table S3).

Exposure was more strongly associated with perceiving the health impacts as entirely bad. Compared with those reporting no exposure, participants reporting local exposure had an 88% higher likelihood of perceiving climate to be entirely bad for people’s health (OR 1.88; 95%CI 1.13, 3.13). For those reporting personal exposure, the odds were higher (OR 1.97; 95%CI 1.06, 3.67) and were further elevated for participants reporting both local and personal exposure (OR 2.53, 95%CI 1.64, 3.89). Being female and having a higher level of education was associated with a greater likelihood of perceiving the health impacts of climate change as being more bad than good or entirely bad (Table S3). 

The model was a good fit with 49.3% correctly predicted, significant (>0.05) for the goodness of fit test, and r^2^ indicating 9.8% of all variance in PIH was accounted for in this model.

## 4. Discussion

The study is based on a survey representative of the UK population. Similar to other social surveys, it relies on participant-reported data and therefore captures participants’ perspectives on climate change and health, together with their reported exposures to flooding and air pollution over the previous 12 months. The proportion of participants reporting flooding to their home in the previous year (6%) is in line with a large national probability survey [29]. While air pollution can be difficult to detect, perceived exposure is associated with measured exposure [31]. In line with this finding, population-weighted estimates suggest that 28% of local authorities in the UK had PM_2.5_ levels above WHO guidelines (annual mean of 10 μgm^−3^) in 2019, the latest year for which data are available [14]. In our survey, 34% of participants reported air pollution/poor air quality in their local area.

Some limitations of our survey design should be noted. Firstly, because potential participants were recruited through an online survey agency, the study excluded those without access to the internet (either via a smartphone or through a connection in their homes). This means that the views and concerns of some of the UK’s most vulnerable populations are likely to be under-represented. The large majority of those without internet access face other forms of social disadvantage [32,33]. However, the COVID pandemic has restricted alternative methods of data collection and prompted a rapid shift toward online data collection [34]. 

Secondly, causality cannot be inferred from cross-sectional surveys. Therefore, it is possible that heightened CCC and PIH resulted in increased awareness of floods and air pollution. However, as noted above, national exposure data are consistent with participant-reported exposure. To increase robustness, we also investigated associations between exposure and CCC and PIH using multinomial regression models that were hierarchical in the design and employed a backwards stepwise approach. This enabled the inclusion of a wide range of potential predictors and the removal of non-significant factors in the final model. Interactions were not investigated; surveys with larger sample sizes may add to the findings presented here. 

Thirdly, while the sample size (*n* = 1024) was similar to or larger than other UK studies [21,22], it prevented more detailed analyses of the patterns and impacts of exposure. For example, pooling smaller ethnic groups into two heterogeneous groups (white; black and minority ethnic groups) may have masked important differences in both exposures and outcomes [35]. A larger sample size would also have enabled separate investigation of floods and air pollution as predictors. 

Fourthly, public perceptions of climate change are known to be influenced by events beyond the individual’s immediate experience, including their exposure to media reporting of climate change events. Climate change coverage in both the mainstream and social media is episodic, peaking at times of scientific and political engagement with climate change [36,37], for example, when major global reports on climate change are published [38,39] and when major global events occur [40]. Our survey was conducted in a month (July 2021) of limited engagement in climate change in the mainstream and social media. We recognize that it is important to repeat the survey at times of heightened media coverage.

## 5. Conclusions

Understanding how the public perceives climate change and its health impacts is essential for climate mitigation and adaptation policies. While studies of populations exposed to extreme weather events and to air pollution have pointed to the importance of direct experience in shaping perceptions of climate change and its health impacts, little is known about the association between exposure and perceptions in the general population. A recent global review [20] located over 50 studies of perceptions of health in the context of climate change in the general population, but less than 10% investigated associations with exposure; of these, none were based in the UK or Europe.

In a representative UK survey of adults, we investigated whether reported exposures were related to public concerns and perceptions. We examined whether people’s awareness of climate change-related exposures in their local area and their experience of these exposures, either personally or among their family and close friends, were associated with climate change concern and perceptions of the health impacts of climate change. In the multivariate analyses, reported exposure to floods and air pollution was associated with heightened CCC and with more negative PIH after controlling for other factors. 

Our findings suggest that policies seeking to increase public awareness of climate concern and its health impacts should pay attention to people’s experiences of climate-related exposures. An appreciation of peoples’ experiences and concerns is increasingly recognised to be essential to securing public support for national and local policies to address climate change and its health impacts [41,42,43]. Our study provides evidence to support this people-centred approach to policy-making. 

## Figures and Tables

**Table 1:** Participant profile (*n* = 1024).

		% (Number)
Age	18–34	30.1 (308)
	35–54	38.6 (395)
	55+	31.3 (321)
Gender *	Male	49.1 (503)
	Female	50.2 (514)
Education (ISCED)	Level 1 (none to GCSE grade D–G)	21.6 (221)
	Level 2 (GCSE grade A–C to higher education qualification)	41.2 (422)
	Level 3 Degree and above	37.2 (381)
Ethnic group **	White	88.2 (903)
	Black and minority ethnic groups	11.8 (121)
Housing tenure	Own home	51.2 (524)
	Rent or other	48.8 (500)
Health status	Good to very good	89.9 (921)
	Fair to very bad	10.1 (103)
Region	Greater London and Southern England	36.3 (372)
	Mid England (West Midlands, East Midlands and East of England)	23.4 (240)
	Northern England (Northwest, Northeast, Yorkshire and the Humber)	24.2 (248)
	Scotland, N. Ireland and Wales	16.0 (164)
Climate change concern	Not at all concerned	4.9 (50)
	Not very concerned	11.8 (150)
	Fairly concerned	47.3 (484)
	Very concerned	36.0 (369)
	Entirely good	4.5 (46)
	More good than bad	5.6 (57)
Impact of climate change on the health of people in the UK ***	Equally good and bad	17.1 (175)
	More bad than good	46.1 (444)
	Entirely bad	25.1 (242)
Reported exposure to flooding	Local	26.9 (275)
	Personal	6.0 (61)
	Family/Friend	11.2 (115)
	Any	336 (344)
Reported exposure to air pollution	Local	33.9 (347)
	Personal	22.9 (234)
	Family/Friend	19.4 (199)
	Any	43.1 (441)

* Response options included ‘prefer to self-define’ (with space provided to do so) and ‘prefer not to share this information’; 7 participants selected one of these options. ** response options included: White—includes any White background; Mixed or multiple ethnic groups—includes White and Black Caribbean, White and Black African, White and Asian, or any other Mixed ethnic group; Asian or Asian British—includes Indian, Pakistani, Bangladeshi, Chinese, or any other Asian background; Black, African, Caribbean, or Black British—includes African, Caribbean, or any other Black background; Other—for example Arab or any other. *** *n* = 60 (5.9%) participants were not this asked this question. When asked a filter question ‘Thinking now about people’s health, which of these statements best describes your views about the impacts of climate change on people’s health in the UK?’, they selected the response ‘Climate change will never have an impact on people’s health in the UK’.

**Table 2:** Climate change concern and perceived health impacts of climate change by reported exposure to floods and air pollution.

		Total		Exposure to Floods and/or Air Pollution				
				None	Local	Personal	Both Local and Personal	Chi^2^ Test
		Count	Column %	Row %	Row %	Row %	Row %	Sig
Climate change concern	Not concerned	171	16.7%	66.1%	16.4%	4.1%	13.5%	<0.001
	Fairly concerned	484	47.3%	42.6%	20.7%	8.7%	28.1%	
	Very concerned	369	36.0%	30.9%	24.4%	7.9%	36.9%	
Impact of climate change on health	Entirely good, more good than bad, equally good and bad	278	28.8%	48.9%	16.5%	9.4%	25.2%	<0.001
	More bad than good	444	46.1%	41.2%	26.8%	4.5%	27.5%	
	Entirely bad	242	25.1%	29.3%	19.4%	11.2%	40.1%	

**Table 3:** Multinomial logistic regression model of reported exposure to floods and air pollution against climate concern.

Climate Change Concern (Reference Category: Not at All/Not Very Concerned)		Sig.	Adjusted OR *	95% CI	
				Lower	Upper
Fairly concerned					
Exposure Reference (no exposure)	Local exposure	0.004	2.070	1.260	3.401
	Personal exposure	0.017	2.827	1.201	6.655
	Both local and personal exposure	<0.001	3.349	1.993	5.629
Very concerned					
Exposure Reference (no exposure)	Local exposure	<0.001	3.398	2.021	5.713
	Personal exposure	0.002	4.114	1.685	10.045
	Both local and personal exposure	<0.001	6.173	3.614	10.545

* Adjusted OR; model adjusted for age, gender, education, health status, country/region of residence. Ethnic group and tenure were inputted into the model but were removed in backwards stepwise approach. Model Fitting—Obs—1017, Log Likelihood 1495.464, Nagelkerke R^2^—0.183, Goodness-of-fit sig. 0.741, Correctly Predicted 53%.

**Table 4:** Multinomial logistic regression of reported exposure to floods and air pollution against perceived impact of climate change on health.

Perceptions of Health Impacts of Climate Change (Reference Category: Entirely Good, More Good than Bad, Equally Bad and Good)		Sig.	Adjusted OR *	95% CI	
				Lower	Upper
Climate change is more bad than good for people’s health					
Exposure Reference (no exposure)	Local exposure	0.004	1.857	1.224	2.816
	Personal exposure	0.072	0.559	0.297	1.053
	Both local and personal exposure	0.191	1.286	0.882	1.876
Climate change is entirely bad than good for people’s health					
Exposure Reference (no exposure)	Local exposure	0.015	1.882	1.130	3.134
	Personal exposure	0.032	1.973	1.060	3.672
	Both local and personal exposure	<0.001	2.526	1.641	3.888

* Adjusted OR; model adjusted for respondents age group, gender, health status, education, region of residence. Age, ethnic group and health status were inputted into the model but were removed in backwards stepwise approach. Model Fitting—Obs—957, Log Likelihood 1604.191, Nagelkerke R^2^—0.098, Goodness-of-fit sig. 0.091, Correctly Predicted 49.3%.

## Data Availability

Under the Privacy Notice accompanying the consent form, participants were advised that only the project team have access to the data and it will not be shared.

## Supplementary Materials

The following are available online at https://www.mdpi.com/article/10.3390/ijerph19042246/s1. Table S1—Participant demographics including gender, age group, and ethnic group along with reported exposure to floods and air pollution plotted against level of climate change concern and also perceived impact of climate change on health. Table S2—Multinomial logistic regression model of reported exposure to floods and air pollution against climate concern. Table S3—Multinomial logistic regression model of reported exposure to floods and air pollution against perceived impact of climate change on health.
