# Supplementary material for: Public Perceptions of Climate Change and Its Health Impacts: Taking Account of People’s Exposure to Floods and Air Pollution
Source: Int J Environ Res Public Health. 2022 Feb 16;19(4):2246. doi: 10.3390/ijerph19042246 (PMC8872106; doi:10.3390/ijerph19042246)
Supplement: Supplementary file 1 [file ijerph-19-02246-s001.zip › ijerph-1584736-supplementary.pdf]

Table S1. Participant demographics including gender, age group and ethnic group along with reported exposure to floods and air pollution plotted against level of climate change concern and also perceived impact of climate change on health.

		Climate change concern								Perceived impact of climate change on health							
		Not at all concerned		Fairly concerned		Very concerned		Total		Entirely good to equally good and bad for people's health		More bad than good for people's health		Entirely bad for people's health		Total	
		Count	Row N %	Count	Row N %	Count	Row N %	Count	P value for Chi²	Count	Row N %	Count	Row N %	Count	Row N %	Count	P value for Chi²
Gender	Male	104	20.7%	224	44.5%	175	34.8%	503		146	31.8%	205	44.7%	108	23.5%	459	
	Female	66	12.8%	257	50.0%	191	37.2%	514	0.003	129	25.9%	238	47.8%	131	26.3%	498	0.126
Age	18-34	44	14.3%	160	51.9%	104	33.8%	308		72	24.7%	133	45.5%	87	29.8%	292	
	35-54	49	12.4%	203	51.4%	143	36.2%	395		122	32.1%	167	43.9%	91	23.9%	380	
	55+	78	24.3%	121	37.7%	122	38.0%	321	<0.001	84	28.8%	144	49.3%	64	21.9%	292	0.085
Tenure	Own	89	17.0%	239	45.6%	196	37.4%	524		135	27.3%	251	50.8%	108	21.9%	494	
	Rent or other	82	16.4%	245	49.0%	173	34.6%	500	0.54	143	30.4%	193	41.1%	134	28.5%	470	0.007
Ethnicity	White	158	17.5%	425	47.1%	320	35.4%	903		244	28.9%	397	47.0%	204	24.1%	845	
	Black & minority ethnic groups	13	10.7%	59	48.8%	49	40.5%	121	0.154	34	28.6%	47	39.5%	38	31.9%	119	0.15
Education	Level 1 - None to GCSE D-G	53	24.0%	111	50.2%	57	25.8%	221		86	42.2%	77	37.7%	41	20.1%	204	
	Level 2 - GCSE A-C to Higher Education Qualification	63	14.9%	204	48.3%	155	36.7%	422		122	30.6%	181	45.4%	96	24.1%	399	
	Level 3 - Degree Level	55	14.4%	169	44.4%	157	41.2%	381	<0.001	70	19.4%	186	51.5%	105	29.1%	361	<0.001
Region	Northern England (North West, North East, Yorkshire & the Humber)	50	20.2%	114	46.0%	84	33.9%	248	<0.001	65	28.1%	108	46.8%	58	25.1%	231	0.163

	Mid England (West Midlands, East Midlands & East of England)	46	19.2%	113	47.1%	81	33.8%	240		63	28.5%	108	48.9%	50	22.6%	221	
	Greater London and Southern England	37	9.9%	176	47.3%	159	42.7%	372		93	26.1%	160	44.9%	103	28.9%	356	
	Scotland, N. Ireland and Wales	38	23.2%	81	49.4%	45	27.4%	164		57	36.5%	68	43.6%	31	19.9%	156	
**Self Reported Health**	**Good/Very Good**	150	16.3%	444	48.2%	327	35.5%	921	0.185	252	29.1%	398	46.0%	215	24.9%	865	793
	**Fair/Bad/Very Bad**	21	20.4%	40	38.8%	42	40.8%	103		26	26.3%	46	46.5%	27	27.3%	99	
**Local flooding**	**No**	142	19.0%	358	47.8%	249	33.2%	749	<0.001	217	31.4%	313	45.3%	161	23.3%	691	0.011
	**Yes**	29	10.5%	126	45.8%	120	43.6%	275		61	22.3%	131	48.0%	81	29.7%	273	
**Local Air Pollution**	**No**	142	21.0%	325	48.0%	210	31.0%	677	<0.001	199	31.7%	290	46.3%	138	22.0%	627	0.002
	**Yes**	29	8.4%	159	45.8%	159	45.8%	347		79	23.4%	154	45.7%	104	30.9%	337	
**Flooding Personal**	**No**	157	17.8%	414	47.0%	310	35.2%	881	<0.001	239	29.0%	399	48.4%	186	22.6%	824	<0.001
	**Yes**	14	9.8%	70	49.0%	59	41.3%	143		39	27.9%	45	32.1%	56	40.0%	140	
**Air Personal**	**No**	151	20.8%	346	47.7%	228	31.4%	725	<0.001	204	30.2%	331	49.0%	140	20.7%	675	<0.001
	**Yes**	20	6.7%	138	46.2%	141	47.2%	299		74	25.6%	113	39.1%	102	35.3%	289	

Table S2. Multinomial logistic regression model of reported exposure to floods and air pollution against climate change concern

Climate change concern: odds ratios (ORs) and 95% confidence intervals (CIs) for climate change concern (reference group: not at all/not very concerned about climate change)			Sig.	OR	95% CI Lower	95% CI Upper
A. Fairly concerned	Intercept		0.465			
	Gender	Reference (Male)				
		Female	0.022	1.574	1.067	2.322
	Age	Reference (55+)				
		18-34	0.001	2.440	1.458	4.082
		35-54	0.000	2.631	1.690	4.098
	Self-reported Health	Reference (Fair/Bad/Very Bad)				
		Good/Very Good	0.046	1.943	1.011	3.375
	Education	Reference (Level 1)				
		Level 2	0.697	1.097	0.689	1.746
		Level 3	0.337	1.261	0.786	2.023
	Region	Reference (Greater London and Southern England)				
		Mid England	0.016	0.527	0.312	0.888
		Scotland, N. Ireland and Wales	0.001	0.377	0.216	0.657
		Northern England	0.002	0.443	0.266	0.739
	Exposure	Reference (No Exposure)				
		Local Exposure	0.004	2.070	1.260	3.401
		Personal Exposure	0.017	2.827	1.201	6.655
		Both Local and Personal	0.000	3.349	1.993	5.629
B. Very concerned	Intercept		0.025			
	Gender	Reference (Male)				
		Female	0.020	1.632	1.082	2.463
	Age	Reference (55+)				
		18-34	0.387	1.274	0.736	2.205
		35-54	0.015	1.781	1.119	2.837
	Self-reported Health	Reference (Fair/Bad/Very Bad)				
		Good/Very Good	0.955	1.020	0.517	2.013
	Education	Reference (Level 1)				
		Level 2	0.073	1.599	0.957	2.674
		Level 3	0.001	.359	1.407	3.954
	Region	Reference (Greater London and Southern England)				

		**Mid England**	0.006	0.465	0.270	0.801
		**Scotland, N. Ireland and Wales**	0.000	0.241	0.132	0.440
		**Northern England**	0.000	0.377	0.221	0.643
	**Exposure**	**Reference (No Exposure)**				
		**Local Exposure**	0.000	3.398	2.021	5.713
		**Personal Exposure**	0.002	4.114	1.685	10.045
		**Both Local and Personal**	0.000	6.173	3.614	10.545
*Adjusted OR; model adjusted for age, gender, education, ethnicity, tenure, health status, country/region of residence. Ethnic group and tenure were inputted into the model but were removed in backwards stepwise approach.						
Model Fitting - Obs - 1017, Log Likelihood 1495.464, Nagelkerke R2 - 0.183, Goodness-of-fit sig. 0.741, Correctly Predicted 53%						

Table S3. Multinomial logistic regression model of reported exposure to floods and air pollution against perceived impact of climate change on health

Climate change concern: odds ratios (ORs) and 95% confidence intervals (CIs) for perceived impact of climate change on health (reference group: Entirely Bad to Equally bad and good Entirely Bad to Equally bad and good			Model 4- S/D, Exposure and PA. Backwards Stepwise			
					95% CI	
			Sig.	OR	Lower	Upper
A. More Bad than Good for People's Health	Intercept		0.162			
	Gender	Reference (Male)				
		Female	0.045	1.379	1.007	1.890
	Tenure	Reference (Rent/Other)				
		Own your home	0.362	0.862	0.627	1.186
	Education	Reference (Level 1)				
		Level 2	0.029	1.553	1.047	2.304
		Level 3	0.000	2.709	1.768	4.151
	Exposure	Reference (No Exposure)				
		Local Exposure	0.004	1.857	1.224	2.816
		Personal Exposure	0.072	0.559	0.297	1.053
		Both Local and Personal	0.191	1.286	0.882	1.876
B. Entirely Bad for Peoples Health	Intercept		0.000			
	Gender	Reference (Male)				
		Female	0.038	1.471	1.022	2.115
	Tenure	Reference (Rent/Other)				
		Own your home	0.098	1.366	0.945	1.976
	Education	Reference (Level 1)				
		Level 2	0.123	1.450	0.904	2.326
		Level 3	0.000	3.162	1.918	5.212
	Exposure	Reference (No Exposure)				
		Local Exposure	0.015	1.882	1.130	3.134
		Personal Exposure	0.032	1.973	1.060	3.672
		Both Local and Personal	0.000	2.526	1.641	3.888

*Adjusted OR; model adjusted for participant's age group, gender, ethnicity, tenure, health status, education, region of residence. Age, ethnic group and health status were inputted into the model but were removed in backwards stepwise approach

Model Fitting - Obs - 957, Log Likelihood 1604.191, Nagelkerke R2 - 0.098, Goodness-of-fit sig. 0.091, Correctly Predicted 49.3%
